# *Abiotrophia defectiva* Infective Endocarditis: A Rare and Dangerous Cause of Endocarditis

**DOI:** 10.1155/2022/7050257

**Published:** 2022-03-08

**Authors:** Ian Lancaster, Deep Patel, Cyrus Tamboli, Patricia Chun, Vikas Sethi, Joseph Namey

**Affiliations:** HCA Healthcare, USF Morsani College of Medicine GME, Largo Medical Center, Largo, FL, USA

## Abstract

Infective endocarditis is an uncommon heart infection, typically involving heart valves. *Abiotrophia defectiva* is a rare cause of endocarditis, typically found within the GI tract, and is usually difficult to isolate and requires specialized media. We report a case of *Abiotrophia defectiva* endocarditis following a root canal.

## 1. Introduction

Infective endocarditis is an infection of a native or prosthetic valve of the heart, mural endocardium, or an indwelling cardiac device [1]. Predisposing risk factors for the development of endocarditis include bicuspid aortic valve, mitral valve prolapse, congenital heart disease, prior infective endocarditis, implanted cardiac device, prosthetic heart valves, IV drug use, chronic kidney disease, malignancy, advanced age, and corticosteroid use [2]. The clinical presentation of infective endocarditis is highly variable and may present as an acute, subacute, or chronic condition reflecting the variable causative micro-organisms, underlying cardiac conditions, and preexisting comorbidities [3]. In patients with infective endocarditis up to 90% of patients are with fevers, night sweats, fatigue, and weight and appetite loss, with approximately 25% having evidence of embolic phenomena at presentation [4]. The modified Duke criteria can be used to help diagnose infective endocarditis [2]. The major criteria include positive blood cultures and echocardiographic evidence of endocarditis (Habib). The minor criteria include the presence of risk factors or intravenous drug use, fever >38°C, vascular phenomena (including arterial emboli, Janeway lesions, and intracranial hemorrhage), immunologic phenome (glomerulonephritis, Osler's nodes, Roth's spots, and rheumatoid factor), and microbiological evidence not consistent with the major criteria [2].

In North America, the most common cause of infective endocarditis is *Staphylococcus aureus* [5]. Other causative organisms of infective endocarditis include viridans streptococci, *Enterococcus* species, *Streptococcus bovis*, and the HACEK organisms (*Haemophilus* species, *Aggregatibacter actinomycetemcomitans*, *Cardiobacterium hominis*, *Eikenella corrodens*, and *Kingella* species) [4]. Other uncommon causes of culture negative endocarditis include *Candida* species, *Bartonella* species, *Chlamydia* species, and *Tropheryma whipplei* [6]. A rare cause of infective endocarditis, *Abiotrophia defectiva*, is a nutritionally variant streptococci found in the oral cavity and intestines [7]. These bacteria are difficult to isolate and requires specialized media for culture, pleomorphic on Gram stain, and can be the cause of culture negative endocarditis [8]. In this case report, we describe the case of a 58-year-old Caucasian female with an unremarkable medical history who had a recent root canal and subsequently developed a case of *Abiotrophia defectiva* endocarditis.

## 2. Case Presentation

The patient was a 58-year-old Caucasian female with a medical history of Hashimoto's thyroiditis and hyperlipidemia who presented to her primary care provider for a weight loss of 26 lbs. over three months. She had noted double vision and feelings of lightheadedness over the last month. She had no fevers, chills, night sweats, shortness of breath, chest pain, or generalized weakness. She was afebrile with normal vital signs and incidentally was found to have developed a new systolic murmur, but an otherwise unremarkable physical exam. On further investigation, she had a root canal performed approximately three months prior to hospitalization. She received no antibiotics prior to the procedure and no indication for antimicrobial agents to be used for prophylaxis.

**Figure 1:**
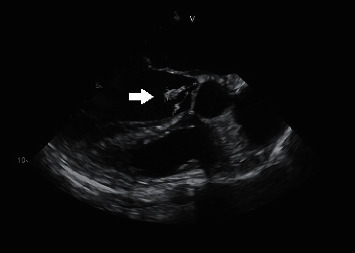
TEE midesophageal long axis view demonstrating mobile vegetation on the aortic valve measuring at 10 mm × 6 mm.

For evaluation of the patient's complaints, a complete metabolic profile, complete blood cell count, coagulation studies, blood cultures, urinalysis with reflex culture, thyroid stimulating hormone, and antinuclear antibody initially demonstrated only mild anemia. Transthoracic echocardiogram was significant for vegetation present on the aortic valve. A transesophageal echocardiogram demonstrated a left ventricular ejection fraction of 60–65%, a medium-sized and pedunculated mobile aortic valve vegetation (Figure 1), as well as a patent foramen ovale.

Additionally, a computed tomography (CT) angiogram was performed and showed severe obstructive coronary artery disease involving the left anterior descending and ramus arteries, true bicuspid aortic valve, and large vegetation measuring at 10 mm × 6 mm.

Magnetic resonance imaging (MRI) brain without contrast, performed due to complaints of double vision, identified three separate 3 mm small acute infarcts involving the right cerebral hemisphere. Magnetic resonance angiogram (MRA) of the neck and brain was negative.

Blood cultures performed on the day of admission were found to be positive in 2 out of 4 bottles for *Abiotrophia* defective, and the patient was subsequently started on gentamicin and penicillin G.

Cardiothoracic surgery was then consulted and performed an aortic valve replacement with a bioprosthetic valve as well as a mitral valve repair. He then remained on penicillin and gentamicin postoperatively for treatment of the *Abiotrophia defectiva* endocarditis and bacteremia. Postoperatively, his course was complicated by sinus node dysfunction/junctional rhythm which ultimately progressed to sick sinus syndrome requiring ventricular pacing, and a dual chamber pacemaker was implanted. Cultures taken from the aortic valve during the procedure were positive for *Abiotrophia defectiva* though Gram stain was unremarkable. Upon discharge, the patient's antibiotics were deescalated from penicillin G every 4 hours and gentamicin every 8 hours to ceftriaxone daily as blood culture sensitivities showed the organism was susceptible to ceftriaxone. The antibiotic was continued for a total of 6 weeks following surgical intervention. At the time of discharge, she was then recommended to follow up with her primary care provider, cardiothoracic surgery team, infectious disease team, and electrophysiologist in the weeks following hospital discharge.

## 3. Discussion

Nutritionally variant streptococci were first described in 1961 and have been isolated from oral, intestinal, and genitourinary flora, as well as blood [9, 10]. A particular subspecies of this, *Abiotrophia defectiva*, has been found to be a rare cause of infective endocarditis, as well as brain abscesses, osteomyelitis, and septic arthritis. It has also been found that up to 11.8% of healthy individuals are colonized with this microbe [11, 12].

*Abiotrophia* species are known for growing in satellite colonies and for their particular nutritional requirements. Though these bacteria can be grown on routine media, as with our patient, growth media enhanced with vitamin B6 or cysteine allow for selective growth [10, 13]. As routine growth media can be used in routine blood cultures, both overgrowth of other bacteria and limited growth due to insufficient nutrients can lead to *Abiotrophia* species being undetectable [10, 13]. With advances in microbiology since the first reported case of this microbe, primary isolation media have changed in composition considerably [10]. In our patient's case, she had positive blood cultures which allowed for targeted antibiotic therapy.

Notably, *Abiotrophia* species and *Granulicatella* species are two rare causes of endocarditis with prior studies noting that endocarditis caused by these micro-organisms is nearly twice as prevalent (1.51% vs 0.88%) as endocarditis caused by the HACEK organisms (*Haemophilus*, *Aggregatibacter*, *Cardiobacterium*, *Eikenella*, and *Kingella*) [14]. Typically, patient's with *Abiotrophia* species endocarditis are younger on average than patients with viridans group streptococci endocarditis by approximately two decades with a mean age of diagnosis of 42 [15]. Dental manipulation has been identified in approximately 32% of cases of *Abiotrophia* defective endocarditis [15], prompting the question of if antibiotic prophylaxis could be justified in this patient population. *Abiotrophia* species have also had a predilection of affecting the mitral valve in cases of endocarditis though the aortic valve has been involved in a minority of cases [16].

Additionally, these patients also have a lower rate of intravenous drug use in comparison to viridans group streptococci endocarditis [15]. In our patient's case, she had a recent history of a root canal 3 months prior to presentation with no history of intravenous drug use.

*Abiotrophia defectiva* endocarditis was previously believed to have a higher mortality rate than viridans group streptococci [14], but recent evaluation of the available data suggests that the mortality rate is similar (approximately 9.2% vs. 9.6%, *p*=0.043) [15]. Of note, there is a higher rate of periannular complications, 28.9% vs. 22%, in comparison to viridans group streptococci, though there has been no evidence of difference greater predilection for prosthetic valves [15]. Interestingly, the rate of cardiac surgical intervention was higher in patients with *Abiotrophia defectiva* endocarditis in comparison to endocarditis caused by viridans group streptococci (65.8% vs. 50%, *p*=0.003) [14, 15].

A study by Bouvet et al. compared different treatment regimens for nonviridans streptococci-induced endocarditis and found that treatment with penicillin and gentamicin was more efficacious than monotherapy with penicillin alone [17]. They also discovered that treatment with vancomycin itself was at least as effective as penicillin plus an aminoglycoside, thereby suggesting that vancomycin would be a viable option in patients showing poor response to the former [17]. This has led to the American Heart Association recommending a regimen of penicillin G and gentamicin for a 4–6-week period with an alternative regimen of vancomycin and gentamicin if the patient is unable to receive penicillin G [18]. Further studies since that time revealed that increasing resistance has emerged towards beta-lactam and macrolide antibiotics, as well as to penicillins [17]. Our patient was started on combined therapy with penicillin G and gentamicin upon initial cultures and responded well. She continued this regimen while hospitalized and was subsequently discharged on ceftriaxone for a simplified antibiotic regimen as blood cultures had shown the microbe was sensitive to the antibiotic.

## 4. Conclusion

Infective endocarditis is an infection of a native or prosthetic of the heart, mural endocardium, or an indwelling cardiac device. Most commonly, infective endocarditis is caused by *Staphylococcus aureus*. *Abiotrophia defectiva* is a rare cause of infective endocarditis and is typically unable to be isolated in blood cultures. It is a common micro-organism found in the gastrointestinal tract and was likely introduced to the blood stream following her root canal. Unique to this patient, his blood cultures were positive, allowing for early identification of the infection and initiation of appropriate antibiotic therapy. Once *Abiotrophia defectiva* is isolated, treatment usually begins with penicillin and gentamicin, and in our patient, he was responsive to this regimen.

## Data Availability

No data were used to support this study.

## References

[1] Cahill T. J., Prendergast B. D. (2016). Infective endocarditis. *The Lancet*.

[2] Habib G., Lancellotti P., Antunes M. J. (2015). 2015 ESC Guidelines for the management of infective endocarditis. *European Heart Journal*.

[3] Rajani R., Klein J. L. (2020). Infective endocarditis: a contemporary update. *Clinical Medicine*.

[4] Murdoch D. R., Corey G. R., Hoen B. (2009). Clinical presentation, etiology, and outcome of infective endocarditis in the 21st century. *Archives of Internal Medicine*.

[5] Chowdhury S., German M. L. (2018). Rare but not infrequent: infective endocarditis caused byAbiotrophia defectiva. *Case Reports in Infectious Diseases*.

[6] Tattevin P., Watt G., Revest M., Arvieux C., Fournier P.-E. (2015). Update on blood culture-negative endocarditis. *Medecine et Maladies Infectieuses*.

[7] Park S., Ann H. W., Ahn J. Y. (2016). A case of infective endocarditis caused byAbiotrophia defectivain korea. *Infection & Chemotherapy*.

[8] Baddour L. M., Wilson W. R., Bayer A. S. (2015). Infective endocarditis in adults: diagnosis, antimicrobial therapy, and management of complications. *Circulation*.

[9] Bouvet A., Villeroy F., Cheng F., Lamesch C., Williamson R., Gutmann L. (1985). Characterization of nutritionally variant streptococci by biochemical tests and penicillin-binding proteins. *Journal of Clinical Microbiology*.

[10] Christensen J. J., Facklam R. R. (2001). Granulicatella and Abiotrophia species from human clinical specimens. *Journal of Clinical Microbiology*.

[11] Angrup A., Gupta P., Agstam S., Manoj R., Kanaujia R., Ray P. (2020). Infective endocarditis caused by Abiotrophia defectiva presenting as anterior mitral leaflet perforation mimicking cleft anterior mitral leaflet. *Journal of Family Medicine and Primary Care*.

[12] Kiernan T. J., O’Flaherty N., Gilmore R. (2008). Abiotrophia defectiva endocarditis and associated hemophagocytic syndrome-a first case report and review of the literature. *International Journal of Infectious Diseases*.

[13] Okada Y., Kitada K., Takagaki M., Ito H.-O., Inoue M. (2000). Endocardiac infectivity and binding to extracellular matrix proteins of oralAbiotrophiaspecies. *FEMS Immunology and Medical Microbiology*.

[14] Téllez A., Ambrosioni J., Llopis J. (2018–2015). Epidemiology, clinical features, and outcome of infective endocarditis due to Abiotrophia species and Granulicatella species: report of 76 cases, 2000-2015. *Clinical Infectious Diseases*.

[15] García-Granja P. E., López J., Vilacosta I. (2018). Nutritionally variant streptococci infective endocarditis: a different view. *Clinical Infectious Diseases*.

[16] Je H., Song D., Chulhun L., Chang (2019). Bacterial endocarditis caused by Abiotrophia defectiva in a healthy adult: a case report with literature review. *Ann Clin Microbiol*.

[17] Bouvet A., Cremieux A. C., Contrepois A., Vallois J. M., Lamesch C., Carbon C. (1985). Comparison of penicillin and vancomycin, individually and in combination with gentamicin and amikacin, in the treatment of experimental endocarditis induced by nutritionally variant streptococci. *Antimicrobial Agents and Chemotherapy*.

[18] Baddour L. M., Wilson W. R., Bayer A. S., Fowler V. G., Bolger A. F., Levison M. E. (2005). Infective endocarditis: diagnosis, antimicrobial therapy, and management of complications: a statement for healthcare professionals from the committee on rheumatic fever, endocarditis, and kawasaki disease, council on cardiovascular disease in the young, and the councils on clinical cardiology, stroke, and cardiovascular surgery and anesthesia, American heart association: endorsed by the infectious diseases society of America. *Circulation*.

